# A metal-polyphenol network-based iron supplement with improved stability and reduced gastrointestinal toxicity for iron deficiency anemia therapy

**DOI:** 10.1016/j.mtbio.2025.101598

**Published:** 2025-02-20

**Authors:** Ying Yao, Yuanzheng Chen, Jie Fu, Jinsong Ding, Wenhu Zhou, Xinyi Chen, Xiuping Wan

**Affiliations:** a Xiangya School of Pharmaceutical Sciences, Central South University, Changsha, Hunan, 410013, China; b Yongkang First People's Hospital of Wenzhou Medical University, Jinhua, 321300, China; c Department of Gastroenterology, The Quzhou Affiliated Hospital of Wenzhou Medical University, Quzhou People's Hospital, Quzhou, 324000, China

**Keywords:** Metal-organic-frameworks, Oral iron supplements, Gut microbiota homeostasis, Oxidative stress, Enteric coating

## Abstract

Iron deficiency anemia (IDA) is a global health concern, particularly affecting women and children of reproductive age. Although oral iron supplements are the standard treatment for IDA, their bioavailability is often compromised by food interactions, and they are associated with significant gastrointestinal side effects. To overcome these limitations, we developed a novel iron nano-supplement, TA-Fe NPs, based on metal-polyphenol networks (MPNs) formed through the coordination of tannic acid (TA) and Fe^3+^. These uniform nanoparticles (∼190 nm) offer enhanced chemical stability and reduced food interference compared to traditional iron supplements. The polyphenolic TA component provides antioxidant properties, effectively mitigating oxidative stress and inflammation induced by free iron ions. To further improve stability and intestinal absorption, TA-Fe NPs were encapsulated in an enteric coating (TA-Fe@L100) to protect against acidic conditions in the stomach. In a mouse model of IDA, TA-Fe@L100 demonstrated superior therapeutic efficacy compared to FeSO_4_, including improvements in hematological parameters, organ iron storage, and gut microbiota balance. Importantly, TA-Fe@L100 alleviated common gastrointestinal side effects associated with iron supplementation, presenting a promising alternative for IDA treatment. Our findings suggest that TA-Fe@L100 is a cost-effective and biocompatible oral iron supplement with minimal side effects, offering significant potential for broader clinical application in the management of IDA.

## Graphical abstract

Iron deficiency anemia (IDA) is a global health issue, especially in women and children of reproductive age. Oral iron supplements are commonly used to treat IDA but have limited bioavailability and cause gastrointestinal side effects. To address these issues, we developed TA-Fe NPs, a novel iron nano-supplement based on metal-polyphenol networks (MPNs) of tannic acid (TA) and Fe^3+^. These nanoparticles (∼190 nm) offer enhanced stability and reduced food interference. The TA component also provides antioxidant benefits, reducing oxidative stress. Encapsulated in an enteric coating (TA-Fe@L100) for improved stability and absorption, TA-Fe@L100 showed superior therapeutic effects in a mouse IDA model compared to FeSO_4_, including better hematological outcomes, iron storage, and gut microbiota balance. Additionally, it reduced gastrointestinal side effects, making it a promising, cost-effective alternative for IDA treatment with minimal side effects.

**Figure:**
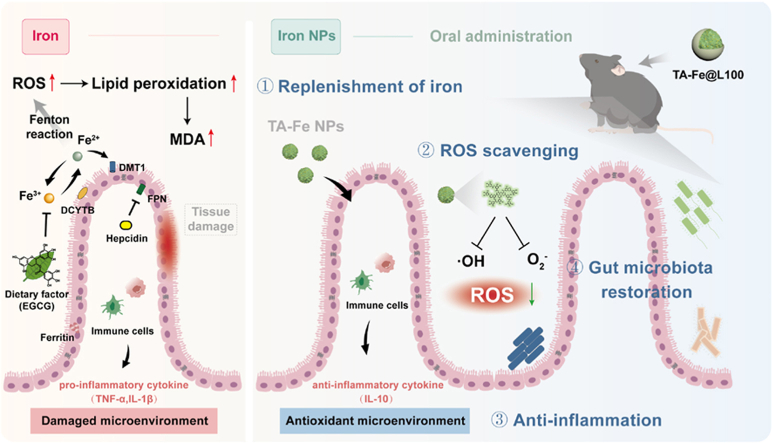

## 1 Introduction

Iron is an essential trace element in the human body, playing pivotal roles in processes such as red blood cell production, oxygen transport and exchange, catalysis of redox reactions, and immune regulation [1]. Maintaining iron homeostasis is crucial for health, and when iron intake fails to meet physiological needs, it can lead to iron deficiency anemia (IDA). IDA is a global health concern, primarily affecting women and children of reproductive age, with approximately a quarter of the global population suffering from this condition [2]. This results in a substantial disease burden, particularly in low- and middle-income countries [3].

The primary cause of IDA is insufficient iron intake, and the most widely used treatment for this condition is oral iron supplementation [4]. Currently, clinical iron supplements are mainly available in the forms of divalent (Fe^2+^) and trivalent (Fe^3+^) iron salts. Divalent iron is absorbed directly by intestinal epithelial cells through the divalent metal transporter-1 (DMT-1) [5,6], while trivalent iron must first be reduced to Fe^2+^ by intestinal reductases before absorption. However, oral iron supplements, particularly in ionic forms, often cause gastrointestinal adverse effects such as nausea, vomiting, constipation, and loss of appetite [7]. These side effects are primarily attributed to the accumulation of free iron in the gastrointestinal tract, where it catalyzes the generation of hydroxyl radicals (•OH) via the Fenton reaction [8,9]. This results in lipid peroxidation, damage to the integrity of intestinal cell membranes, and oxidative injury to the gastrointestinal mucosa [10]. Moreover, the absorption of free iron is affected by the presence of food, which may lead to iron-chelation and reduced bioavailability [11]. In response to these challenges, second-generation iron supplements such as ferrous lactate and ferrous citrate, which release iron ions more slowly, have been developed to mitigate these side effects. Additionally, third-generation chelated iron supplements, such as Ferric Maltol (developed by Shield Therapeutics), aim to improve bioavailability by absorbing iron in molecular form [12,13]. However, the clinical effectiveness of these formulations remains suboptimal, and their higher costs limit their accessibility. Therefore, the development of new oral iron supplements with improved efficacy, minimal side effects, and cost-effectiveness is a significant unmet need.

With advances in nanomedicine, researchers have explored the potential of nanomaterials to encapsulate iron salts or develop iron composite nanoparticles. These nanoparticle-based formulations aim to enhance iron absorption, reduce gastrointestinal irritation, and improve bioavailability [14]. Nanoformulations of iron can mitigate gastrointestinal discomfort by slowly releasing iron ions or through the overall absorption of the nanoparticles [15,16]. Furthermore, the biological properties of the nanoparticle carrier can help alleviate the toxic effects of iron. However, research on oral nano-iron supplements is still in its early stages, with challenges such as complex preparation methods, high production costs, and limited iron loading capacity. Additionally, the efficacy of current nanoparticle-based iron supplements in reducing gastrointestinal side effects remains limited.

To address these issues, this study developed a novel nano-iron supplement based on metal-organic frameworks (MOFs) for the treatment of IDA. MOFs are materials formed by the self-assembly of metal ions and organic ligands through coordination bonds, offering simple synthesis, low cost, and scalability for industrial production [17]. MOFs formed with polyphenols as organic ligands are referred to as metal-polyphenol networks (MPNs). These MPNs exhibit excellent biocompatibility, high drug loading capacities, and have been widely used in drug delivery applications [18,19]. Polyphenols like tannic acid (TA) and epigallocatechin gallate (EGCG) not only form stable MPNs with metal ions but also possess a broad spectrum of pharmacological activities that can enhance therapeutic efficacy [[20], [21], [22], [23], [24], [25]]. Therefore, MPNs have been employed for delivering organic small molecules, proteins, nucleic acids, and even living organisms, such as bacteria and cells [[26], [27], [28], [29], [30]]. However, there are no reports to date on the use of MPNs as inorganic metal carriers for metal element supplementation.

**Scheme 1:**
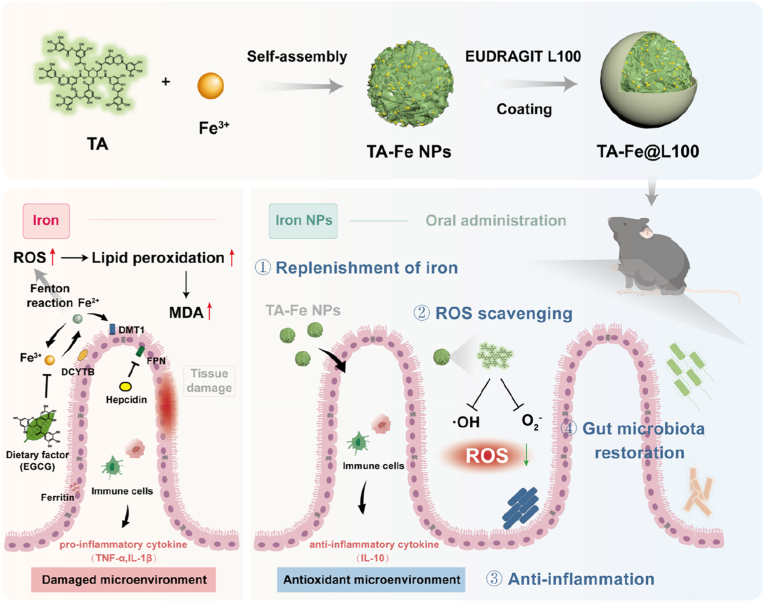
Schematic representation of the synthesis of TA-Fe@L100 and its application advantages in IDA treatment. Iron supplements in their ionic form are absorbed via DMT-1, but their absorption can be hindered by food interactions, such as chelation with polyphenols. Moreover, free iron ions promote the Fenton reaction, generating free radicals that induce oxidative stress and gastrointestinal inflammation. In contrast, TA-Fe@L100 is absorbed as nanoparticles, bypassing food-induced interactions and avoiding the Fenton reaction. The TA component within the nanoparticles provides broad-spectrum free radical scavenging, reducing oxidative damage and local inflammation. Additionally, TA-Fe@L100 regulates gut microbiota homeostasis, offering a multifaceted therapeutic approach for IDA.

In this study, we developed a novel MPN-based iron ion nano-delivery system, TA-Fe NPs, through the self-assembly of TA and Fe^3+^ (Scheme 1). TA-Fe NPs are uniform nanoparticles with an average size of approximately 190 nm, demonstrating chemical stability and effective cellular uptake as whole nanoparticles. The absorption of TA-Fe NPs is not influenced by food, and the polyphenolic TA component imparts strong antioxidant properties, reducing the intracellular reactive oxygen species (ROS) levels, alleviating oxidative stress, and minimizing local inflammation. To prevent structural damage from gastric pH variations, TA-Fe NPs are coated with an enteric polymer (TA-Fe@L100), allowing for improved stability during transit through the gastrointestinal tract. Compared to traditional FeSO_4_ supplementation, TA-Fe@L100 demonstrated enhanced in vivo efficacy in a mouse model of IDA, improving hematological parameters, organ iron storage, and gut microbiota balance. Crucially, TA-Fe@L100 was shown to reduce gastrointestinal side effects and maintain excellent biocompatibility, supporting its potential as a promising new oral iron supplement.

## 2 Materials and methods

### 2.1 Materials

Tannic acid (TA) and ferrous sulfate heptahydrate (FeSO_4_·7H_2_O) were purchased from Macklin Biochemical Co., Ltd. (Shanghai, China). Ferric trichloride hexahydrate (FeCl_3_·6H_2_O) and DCFH-DA were acquired from Sinopharm Chemical Reagent Co., Ltd. (Shanghai, China). EUDRAGIT L100 was sourced from Evonik Industries (Germany). DAPI was procured from Solarbio Science & Technology Co., Ltd. (Beijing, China). CCK8 was purchased from APExBIO (USA). DPPH and ABTS were obtained from Med Chem Express (MCE, USA) and BioTechne (Shanghai, China). MEM medium was purchased from PNS Life Science Co., Ltd. (Wuhan, China). 0.25 % EDTA trypsin was sourced from Gibco Life Technologies (USA). VivaCell fetal bovine serum was purchased from XB Biotech Co., Ltd. (Shanghai, China). Antibodies for TNF-α, IL-1β, and IL-1β were acquired from Abcam Biotech Co., Ltd. (Wuhan, China).

### 2.2 Cell culture

Caco-2 cells were purchased from iCell Bioscience Inc. (Shanghai, China). The cells were cultured in MEM medium supplemented with 10 % FBS, 1 % penicillin (50 U/mL), and streptomycin (50 U/mL) in a 5 % CO_2_ atmosphere at 37 °C.

### 2.3 Animals

Healthy male C57BL/6 mice (4 weeks old, 20 ± 2 g) were purchased from Hunan SJA Laboratory Animal Co., Ltd. (Changsha, China), and were maintained in an SPF environment. All animal experiment protocols were approved by the Experimental Animal Ethics Committee of Hunan Evidence-Based Biotechnology Co., Ltd. Ethical approval number: 2024XZ034.

### 2.4 Synthesis and characterization of TA-Fe NPs

TA was dissolved in ultrapure water to prepare a solution containing 40 mg/mL, and FeCl_3_·6H_2_O was dissolved in ultrapure water to prepare a solution containing 10 mg/mL 800 μL of TA solution was added to a beaker containing 50 mL ultrapure water. 800 μL of FeCl_3_·6H_2_O solution was added dropwise under stirring at room temperature for 5 min. The reaction mixture was centrifuged at 16000 rpm for 20 min, and the precipitate was washed twice and dispersed in 2 mL ultrapure water for storage at 4 °C. To prepare red fluorescent TA-Fe NPs, 500 μL ultrapure water was replaced with 1 mg/mL Rhodamine B solution and the same procedure was followed. Rhodamine B is initially introduced into the aqueous phase, followed by the addition of the raw materials for nanoparticle synthesis. Rhodamine B binds to the nanoparticles via electrostatic interactions and hydrogen bonding between its carbonyl group and the hydroxyl groups of tannic acid. Encapsulation efficiency and drug loading of TA-Fe NPs were measured using ICP-OES (Agilent). Hydrodynamic size, PDI, and ζ potential were determined using a Malvern Zeta Sizer Nano Series (Nano ZS, Malvern, UK). Stability was assessed by measuring size and PDI changes over 72 h in different media. Morphology and elemental composition were observed with TEM-EDS (Titan G2 60-30, FEI, USA). Surface chemical composition was analyzed by XPS (NEXSA, Thermo Fisher, USA). UV–vis and FTIR spectra were obtained using a UV-2600 spectrophotometer (Shimadzu) and FTIR (Bruker, Germany). Particle size at different pH levels (1.4, 3, 5, 6.8) was measured with a Malvern Nanoparticle Size Zeta Potential Analyzer at 25 °C.

### 2.5 Cellular uptake study

Caco-2 cells were seeded in 24-well plates (1 × 10^5^ cells/well) and incubated overnight. Rhodamine B-labeled TA-Fe NPs (100 μM) was added and incubated for 1, 2, and 4 h. Subsequently, the cells were observed under fluorescence microscope.

For uptake mechanism study, cells were pre-incubated with PBS (control), chlorpromazine (10 μg/mL), amiloride (10 μg/mL), nystatin (15 μg/mL), or sodium azide (1 mg/mL) for 30 min, followed by incubation with Rhodamine B-labeled TA-Fe NPs (100 μM) for 4 h. Subsequently, the cells were observed under fluorescence microscope.

To assess the effect of placement time on iron stability and iron uptake, a bivalent iron content assay kit (Boxbio, Beijing, China) was used to determine the content of bivalent iron in FeSO_4_ and TA Fe NPs solutions after fresh configuration and placement for 7 days. Then, Caco-2 cells were seeded in 10 cm dishes (2 × 10^6^ cells/dish) and incubated overnight. Freshly prepared and 7-day-old FeSO_4_ (pre-centrifuged to remove precipitate) were added and incubated for 24 h. The cells were collected and digested with HCl. The iron content was determined by ICP-OES (Agilent). The freshly prepared and 7-day-old TA-Fe NPs were treated as described above.

To study the effect of dietary factors on iron uptake, FeSO_4_ and TA-Fe NPs were pre-incubated with EGCG at various concentrations for 1 h, then incubated with Caco-2 cells for 24 h. The cells were collected and digested with HCl. The iron content was determined by ICP-OES (Agilent).

### 2.6 DPPH radical scavenging activity

Different concentrations of TA-Fe NPs (20 μL) were mixed with DPPH solution (500 μL, 0.3 mM) to achieve final concentrations of 0, 5, 10, 25, 50, and 75 μM. The mixture was incubated in the dark, and absorbance at 517 nm was measured every 5 min for 30 min. After incubation, UV spectra (400–800 nm) were recorded to determine the DPPH scavenging activity at each concentration.

### 2.7 ABTS radical scavenging activity

ABTS solution (7.4 mM) was mixed with (NH_4_)_2_S_2_O_8_ solution (2.6 mM) in a 1:1 ratio (v:v) and incubated in the dark at 4 °C overnight to generate ABTS^+^• solution. Different concentrations of TA-Fe NPs (200 μL) were mixed with ABTS^+^• solution (800 μL) to achieve final concentrations of 0, 5, 10, 25, 50, 75, and 100 μM. The mixture was incubated in the dark, and absorbance at 734 nm was measured every 5 min for 30 min. After incubation, UV spectra (500–900 nm) were recorded to determine the ABTS^+^• scavenging activity at each concentration.

### 2.8 •OH and O_2_^−^• radical scavenging activity

The scavenging activities of TA-Fe NPs on •OH and O_2_^−^• at different concentrations were measured using hydroxyl radical (•OH) assay kits and superoxide anion radical (O_2_^−^•) assay kits (Nanjing Jiancheng Bioengineering Institute, China).

### 2.9 Cytotoxicity evaluation

Cell viability was assessed in vitro using the CCK-8 assay. Caco-2 cells were seeded in 96-well plates (5 × 10^3^ cells/well) and incubated overnight. Gradient concentrations of FeSO_4_ and TA-Fe NPs were added and incubated for 24 h. Subsequently, cell viability was evaluated using the CCK-8 assay.

### 2.10 Detection of intracellular ROS and LPO levels

Caco-2 cells were seeded in 24-well plates (1 × 10^5^ cells/well) and incubated overnight. Different concentrations of FeSO_4_ and TA-Fe NPs were added and incubated for 24 h. After removing the drugs, cells were washed with PBS and incubated with DCFH-DA (10 μM) or C11-BODIPY (10 μM) for 30 min. Fluorescence intensity was observed under a microscope after washing.

### 2.11 Detection of intracellular MDA levels

Caco-2 cells were seeded in 10 cm dishes (2 × 10^6^ cells/dish) and incubated overnight. FeSO_4_ and TA-Fe NPs (1 mM) were added and incubated for 24 h. The cells were collected and lysed to extract MDA. MDA content was measured according to the protocol of the MDA assay kit (Langjie Ke Technology Co., Ltd., Beijing, China). Total protein concentration was quantified using a BCA Protein Assay Kit (MCE, Shanghai, China) for normalization of cell numbers.

### 2.12 Synthesis and characterization of TA-Fe@L100

After high-speed centrifugation, the TA-Fe NPs precipitate was redissolved in anhydrous ethanol. EUDRAGIT L100 was dissolved in equal volume of anhydrous ethanol at a 1:5 mass ratio with TA-Fe NPs. The mixture was stirred for 5 min, and ethanol was removed by rotary evaporation. The solid was vacuum-dried for 24 h and ground to obtain enteric-coated TA-Fe NPs. To prepare simulated gastric fluid (SGF), 234 mL concentrated HCl was diluted to 1000 mL with water, then mixed with 10 g pepsin and diluted to 1000 mL. For simulated intestinal fluid (SIF), 6.8 g KH_2_PO_4_ was dissolved in 50 mL water, pH adjusted to 6.8 with 0.1 M NaOH, and 10 g pancreatin was dissolved in water, then combined and diluted to 1000 mL. The hydrated particle size of TA-Fe@L100 was measured in SGF (pH 1.4) and SIF (pH 6.8) at 25 °C. Then, TA-Fe@L100 was incubated in SGF (pH 1.4) and SIF (pH 6.8) fluids for 4 and 8 h, respectively, then freeze-dried. Morphological observation was done by SEM, and the crystal structure was analyzed by XRD. For in vitro release studies, TA-Fe NPs and TA-Fe@L100 were dispersed in solutions of pH 1.4, pH 3, pH 5, and pH 6.8. Samples were collected at 0, 0.5, 1, 1.5, 2 h, centrifuged, and iron content in the supernatant was measured by ICP-OES to determine the release profile.

### 2.13 Establishment of iron deficiency anemia mouse model

Four-week-old male C57BL/6 mice were used to establish the IDA model. The normal group was fed a standard diet, and the model group received an iron-deficient diet with tail vein bleeding (10 drops/time, twice/week). Hemoglobin levels were monitored weekly. The IDA model was successfully established when hemoglobin levels fell below 125 g/L, and the iron-deficient diet was continued thereafter.

### 2.14 Animal experiments

Iron deficiency anemia mice were randomly divided into five groups (n = 3), with one group as Model group and the remaining four as treatment groups. The Control and Model groups were gavaged with saline daily. The treatment groups were orally administered FeSO_4_ (1 mg/kg), FeSO_4_ (3 mg/kg), TA-Fe@L100 (1 mg/kg), and TA-Fe@L100 (3 mg/kg), respectively. The treatment was administered continuously for 5 weeks, with weekly monitoring of the mice's body weight.

### 2.15 Blood parameter detection

Whole blood was collected from mice (with heparin) and analyzed by hematology analyzer for red blood cell count (RBC), hemoglobin (HB), hematocrit (HCT), and mean corpuscular volume (MCV). ALT and AST levels in serum were measured using an automatic biochemical analyzer.

### 2.16 Biochemical analysis

Liver tissue was collected at the end of treatment. The content of liver ferritin was measured by ferritin detection kit (Walker Biotechnology Co., Ltd., Zhengzhou, China). Total RNA was extracted from the liver. The relative expression level of hepcidin mRNA was determined by SYBR Green PCR fluorescent detection kit (Vazyme Biotech Co., Ltd., Nanjing, China). Plasma SOD levels and MDA contents in the stomach and small intestine were measured using SOD and MDA assay kits (Boxbio Technology Co., Ltd., Beijing, China).

### 2.17 Analysis of the gut microbiota

At the end of the administration, mouse feces were collected for total DNA extraction. 16S rRNA sequencing was used to investigate the relative richness of gut microbiota in different groups.

### 2.18 Histological analysis

Major organs were preserved with 4 % paraformaldehyde fixation, paraffin-embedded, and processed for hematoxylin and eosin (H&E) staining.

### 2.19 Immunofluorescence staining

Small intestinal tissue sections were stained overnight with TNF-α, IL-1β, and IL-10 antibodies. Fluorescently labeled secondary antibodies were subsequently added and incubated for 1 h. After the nuclei were stained with DAPI, the sections were observed under a fluorescence microscope.

### 2.20 Statistical analysis

Data were presented as mean ± standard deviation (SD). GraphPad Prism 8.0 was used for statistical analysis. Two samples were compared by *t*-test, and multiple samples were compared by One-way ANOVA. ∗p < 0.05, ∗∗p < 0.01, ∗∗∗p < 0.001, ∗∗∗∗p < 0.0001.

## 3 Results and discussions

### 3.1 Preparation and characterization of TA-Fe NPs

**Fig. 1:**
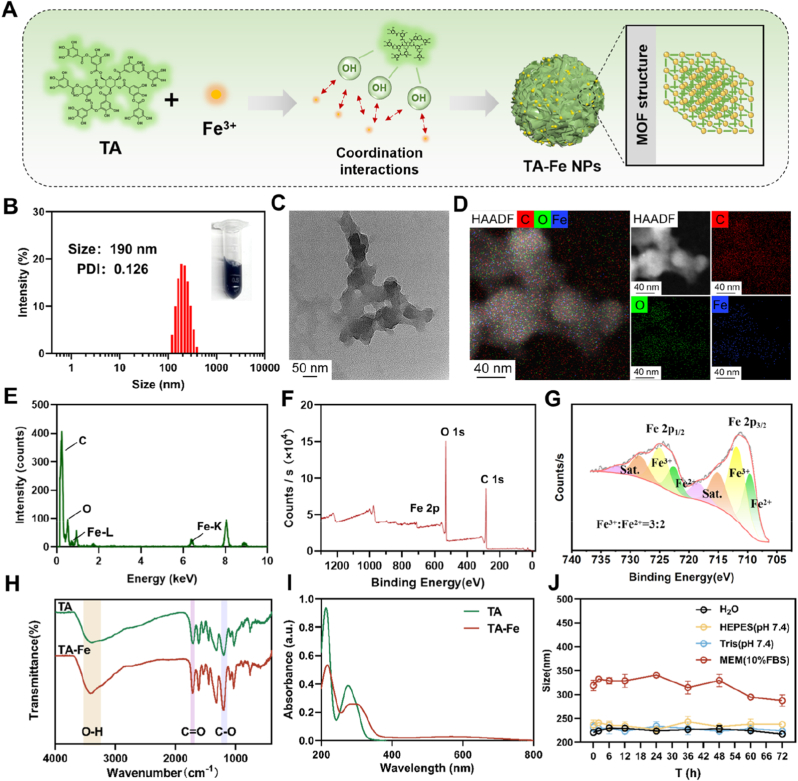
(A) Schematic representation of the synthesis process of TA-Fe NPs. (B) Hydrated particle size distribution of TA-Fe NPs, with an inset showing the physical appearance of the sample. (C) TEM images of TA-Fe NPs. (D) Elemental mapping images and (E) EDS spectrum of TA-Fe NPs. (F) XPS spectra of TA-Fe NPs, including (G) Fe peak fitting results. (H) Infrared spectra of TA and TA-Fe NPs. (I) UV–Vis absorption spectra of TA and TA-Fe NPs. (J) Colloidal stability of TA-Fe NPs in various media over 72 h (n = 3).

TA-Fe nanoparticles (TA-Fe NPs) were self-assembled from tannic acid (TA) and iron ions (Fe^3+^) in aqueous solution. The multiple phenolic hydroxyl groups in TA coordinate with Fe^3+^ to form a stable metal-organic framework (MOF) structure (Fig. 1A). During the reaction, the solution's color changed from light yellow to blue-black (Fig. S1), confirming successful coordination between TA and Fe^3+^. The resulting TA-Fe NPs had a hydrated particle size of approximately 190 nm with a uniform size distribution (PDI <0.2) (Fig. 1B). The zeta potential of the nanoparticles was measured as −28 mV, attributed to the dissociation of phenolic hydroxyl groups in aqueous solution, leading to a negatively charged surface.

The assembly mechanism of TA and Fe^3+^ was investigated through REST (Replica Exchange with Solute Tempering) molecular dynamics simulations (Fig. S2A). Cluster analysis of major conformations revealed that TA molecules interact predominantly through molecular nesting and close packing. Upon Fe^3+^ addition, the structural integrity of the TA composite was maintained, with Fe^3+^ forming a stable ternary complex by coordinating with three phenolic hydroxyl groups from two TA molecules (Fig. S2B). TA molecules interacted via hydrogen bonding between phenolic hydroxyl groups and π–π stacking between benzene rings, exhibiting a binding energy of −50.147 kcal/mol. With Fe^3+^ incorporation, the binding energy increased to −70.836 kcal/mol, highlighting stronger interactions within the composite (Fig. S2C). Fe^3+^ also significantly altered the LUMO and HOMO orbitals of TA, reflecting its impact on phenolic hydroxyl groups (Fig. S2D).

Transmission electron microscopy (TEM) revealed that TA-Fe NPs had an irregular spherical morphology with uniformly distributed C, O, and Fe elements (Fig. 1C and D). Energy-dispersive X-ray spectroscopy (EDS) confirmed the presence of these elements (Fig. 1E). X-ray photoelectron spectroscopy (XPS) identified characteristic peaks for Fe^3+^ at 711.79 eV and 724.95 eV, and for Fe^2+^ at 709.55 eV and 722.57 eV, with satellite peaks observed at 715.03 eV, 718.41 eV, 728.38 eV, and 732.08 eV (Fig. 1F and G). The Fe element existed in both Fe^3+^ and Fe^2+^ forms with a calculated molar ratio of approximately 3:2.

Infrared (IR) spectroscopy showed broad absorption bands at 3400–3200 cm^−1^, indicating phenolic hydroxyl group vibrations and intermolecular hydrogen bonding. Additional characteristic peaks included carbonyl (C=O) stretching at 1700 cm^−1^, aromatic ring (C=C) vibrations at 1600–1400 cm^−1^, and carbon-oxygen (C–O) stretching of ether and ester bonds at 1200 cm^−1^ (Fig. 1H). UV–Vis spectroscopy revealed TA's characteristic peaks at 276 nm and 213 nm [31], which exhibited a red shift in TA-Fe NPs due to coordination-induced changes in the π–π∗ and n–π∗ transitions (Fig. 1I). Furthermore, TA-Fe NPs showed consistent particle size after 72 h of incubation in ultrapure water, HEPES buffer (pH 7.4), Tris buffer (pH 7.4), and MEM containing 10 % fetal bovine serum, demonstrating excellent colloidal stability (Fig. 1J).

### 3.2 Cellular uptake of TA-Fe NPs and its influencing factors

**Fig. 2:**
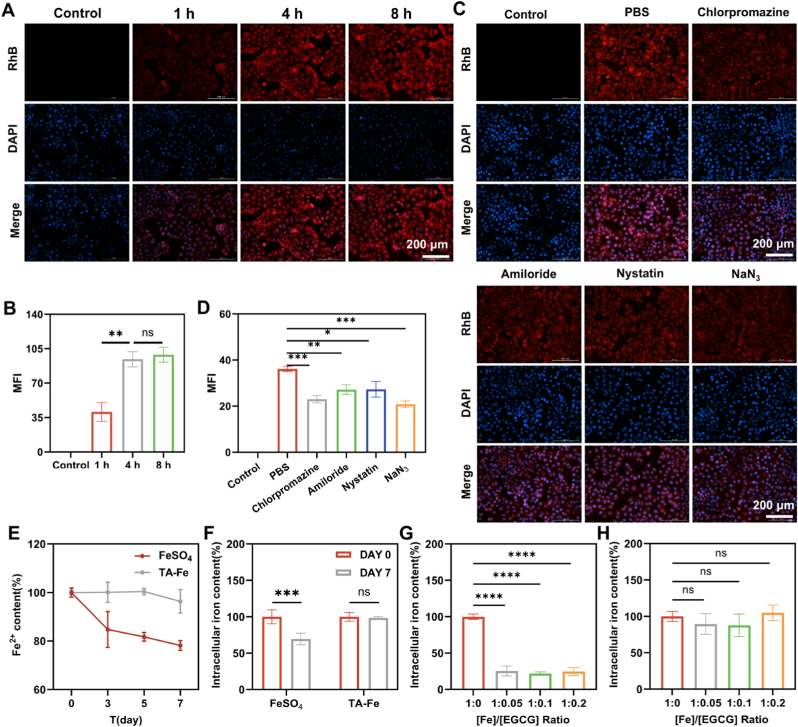
(A) Time-dependent cellular uptake of TA-Fe NPs observed by fluorescence microscopy. (B) Quantification of fluorescence intensity in (A) (n = 3). (C) Cellular uptake of TA-Fe NPs in the presence of various endocytosis inhibitors. (D) Quantification of fluorescence intensity in (C) (n = 3). (E) Changes in Fe^2+^ content in FeSO_4_ and TA-Fe NPs stored at room temperature for 7 days (n = 3). (F) Cellular uptake of FeSO_4_ and TA-Fe NPs after 0 and 7 days of storage (n = 3). (G) Cellular iron uptake from FeSO_4_ at different Fe/EGCG ratios (n = 3). (H) Cellular iron uptake from TA-Fe NPs at different Fe/EGCG ratios (n = 3).

The absorption of TA-Fe nanoparticles (TA-Fe NPs) by intestinal cells is critical for their efficacy as iron supplements. To simulate in vivo absorption, we conducted cellular uptake experiments. TA-Fe NPs were labeled with Rhodamine B (red fluorescence) for visualization, while nuclei were stained with DAPI (blue fluorescence). Fluorescence microscopy revealed that the intracellular red fluorescence signal increased over time and reached saturation within 4 h, indicating rapid and efficient nanoparticle uptake (Fig. 2A and B). To elucidate the cellular uptake mechanism, various inhibitors were employed: chlorpromazine (clathrin-mediated endocytosis inhibitor), amiloride (macropinocytosis inhibitor), nystatin (caveolin-mediated endocytosis inhibitor), and sodium azide (inhibitor of ATP-dependent processes). All inhibitors significantly reduced nanoparticle uptake, suggesting that the cellular internalization of TA-Fe NPs occurs via multiple endocytosis pathways (Fig. 2C and D).

The absorption of inorganic iron supplements is influenced by chemical stability and interactions with dietary components [11].For example, Fe^2+^ oxidizes to Fe^3+^ in aqueous solutions, forming insoluble iron hydroxide precipitates, which reduces absorption efficiency [32]. After 7 days of storage, the Fe^2+^ content in FeSO_4_ solution decreased by 22 %, accompanied by a 40 % reduction in cellular uptake efficiency (Fig. 2E and F). In contrast, the Fe^2+^ content and cellular uptake efficiency of TA-Fe NPs remained unchanged, indicating that encapsulating iron ions in the TA framework significantly enhances chemical stability. Dietary polyphenols, such as epigallocatechin gallate (EGCG) found in tea and other foods, strongly chelate free iron, reducing bioavailability. Using EGCG as a model polyphenol, we observed that a Fe/EGCG molar ratio of 1:0.05 reduced cellular iron uptake from FeSO_4_ by 75 % (Fig. 2G). In contrast, the uptake of TA-Fe NPs was unaffected by EGCG across the tested concentrations (Fig. 2H). These results demonstrate that the structural fixation of iron in TA-Fe NPs enhances chemical stability and mitigates interactions with dietary components, ensuring stable oral bioavailability and consistent efficacy.

### 3.3 Anti-oxidative activities of TA-Fe NPs

**Fig. 3:**
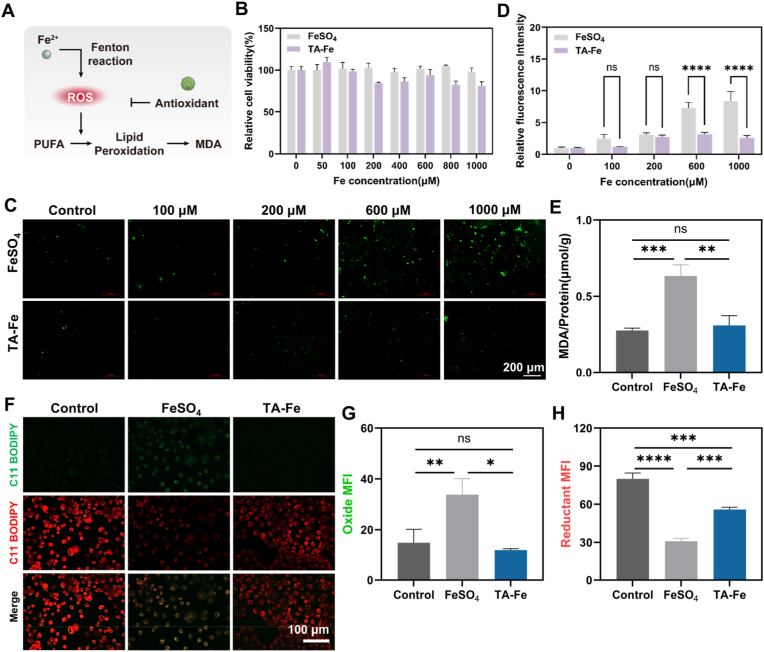
(A) Molecular mechanism of TA-Fe NPs in antioxidation and cell protection. (B) Cytotoxicity of TA-Fe NPs and FeSO_4_ at different concentrations on Caco-2 cells (n = 3). (C) ROS levels in Caco-2 cells after treatment with varying concentrations of TA-Fe NPs and FeSO_4_. (D) Semi-quantitative analysis of ROS fluorescence intensity in (C) (n = 3). (E) Intracellular MDA levels in Caco-2 cells after treatment with TA-Fe NPs and FeSO_4_ (n = 3). (F) LPO levels in Caco-2 cells measured using the C11-BODIPY probe after treatment with TA-Fe NPs and FeSO_4_. Semi-quantitative analysis of (G) oxidized fluorescence and (H) reduced fluorescence in (F) (n = 3).

A major side effect of oral iron salts as supplements is gastrointestinal irritation caused by reactive oxygen species (ROS) generated via the Fenton reaction of iron ions [8].ROS can trigger lipid peroxidation, compromise intestinal cell membrane integrity, and damage gastrointestinal mucosa [10].Addressing gastrointestinal ROS is thus an effective strategy to alleviate these adverse effects [[33], [34], [35], [36]]. In this study, TA was chosen as the organic ligand for iron ions not only for its chelating ability but also for its natural polyphenolic antioxidant properties, which could potentially mitigate the oxidative damage associated with iron ion supplementation (Fig. 3A) [37]. To verify this hypothesis, we assessed the ROS-scavenging ability of TA-Fe NPs in both in vitro and cellular settings.

At the in vitro level, we used 2,2-diphenyl-1-picrylhydrazyl (DPPH) to evaluate free radical scavenging activity. DPPH exhibits a stable purple color with a UV absorption peak at 517 nm. After 30 min of co-incubation with TA-Fe NPs at different concentrations, the absorption intensity at 517 nm decreased in a concentration-dependent manner (Fig. S3A). The DPPH scavenging kinetics revealed rapid clearance within minutes (Fig. S3B). As the concentration of TA-Fe NPs increased from 5 μM to 75 μM, the solution color shifted from purple to yellow, and the DPPH scavenging rate increased from 10.34 % to 66.64 % (Fig. S3C). Furthermore, TA-Fe NPs demonstrated concentration-dependent scavenging of various radicals, including ABTS^+^•, •OH, and O_2_^−^•, confirming their broad-spectrum free radical scavenging capability (Figs. S3D–J).

At the cellular level, the biocompatibility of TA-Fe NPs was first evaluated using the CCK-8 assay. After 24 h of co-incubation with Caco-2 cells, cell viability remained above 80 %, confirming good biocompatibility (Fig. 3B). Intracellular ROS levels were assessed using the DCFH-DA probe (Fig. 3C). While FeSO_4_ treatment caused a concentration-dependent increase in ROS levels, TA-Fe NPs showed no significant intracellular ROS signal. Quantitative analysis revealed that at an equivalent iron concentration of 1 mM, the ROS signal intensity for TA-Fe NPs was 60 % lower than that of FeSO_4_ (Fig. 3D), attributed to the antioxidant activity of TA within the nanoparticle structure.

Lipid peroxidation, a direct consequence of ROS accumulation, leads to the production of lipid peroxides (LPO) and compromises cell membrane integrity [38]. Malondialdehyde (MDA) levels were measured as an indicator of lipid peroxidation. FeSO_4_ treatment significantly increased intracellular MDA levels, indicating membrane damage (Fig. 3E). In contrast, no significant MDA changes were observed in the TA-Fe NP-treated group, highlighting their protective effect against lipid peroxidation. Further analysis using the C11-BODIPY fluorescent probe to detect intracellular LPO revealed that FeSO_4_ caused a shift in fluorescence from red (590 nm) to green (510 nm), reflecting increased lipid peroxidation. TA-Fe NPs maintained low green fluorescence levels, indicating reduced lipid peroxidation (Fig. 3F–H). These results demonstrate that TA-Fe NPs, through their antioxidant properties, effectively alleviate iron-induced oxidative stress and reduce the potential toxic side effects of oral iron supplementation.

### 3.4 Preparation and characterization of TA-Fe@L100

After entering the gastrointestinal tract (GI tract), oral medications undergo complex physiological environments, especially drastic pH changes, rapidly rising from the acidic environment of the stomach (pH about 1.4) to the neutral environment of the small intestine (pH about 6.8). To clarify the stability of nanoparticles during gastrointestinal transport, the appearance and particle size changes of TA-Fe NPs were measured under different pH conditions (Fig. S4). Under the neutral pH conditions of simulated intestinal fluid, the colloidal stability of TA-Fe NPs is good, and there is no significant change in particle size and appearance. However, as the pH value decreases, the stability of TA-Fe NPs significantly decreases. Under pH 1.4 conditions, the particles quickly aggregate and the nanoparticles turn from blue black to clear. The above results indicate that TA-Fe NPs are prone to degradation under acidic conditions, which may be due to protonation of the phenolic hydroxyl groups in the TA structure, leading to a weakened coordination ability with iron ions [39,40]. Therefore, it is necessary to enhance the anti-acid degradation ability of TA-Fe NPs during oral delivery.

**Fig. 4:**
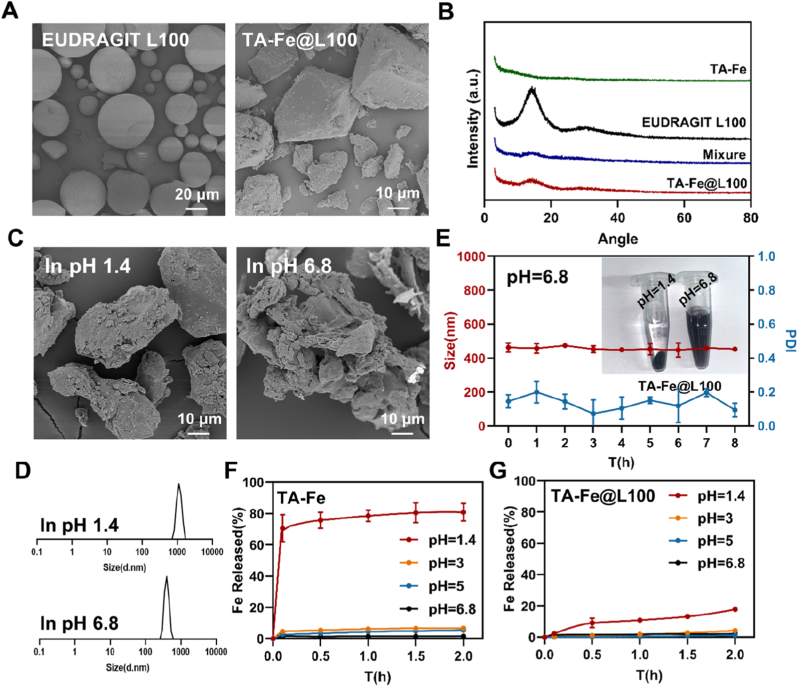
(A) SEM images of EUDRAGIT L100 and TA-Fe@L100. (B) XRD patterns of TA-Fe, EUDRAGIT L100, the mixture of TA-Fe + EUDRAGIT L100, and TA-Fe@L100. The (C) surface morphology and (D) particle size distribution of TA-Fe@L100 after incubation at pH 1.4 and 6.8. (E) The colloidal stability of TA-Fe@L100 over 8-h in SIF (n = 3). Inset: Appearance of TA-Fe@L100 in SGF and SIF. (F, G) The Fe release curves of TA-Fe and TA-Fe@L100 under different pH conditions (n = 3).

To improve the gastric stability of TA-Fe NPs, we encapsulated TA-Fe NPs in enteric coated material EUDRAGIT L100 and constructed an enteric coated formulation TA-Fe@L100. Through scanning electron microscopy (SEM) observation, it was found that EUDRAGIT L100 is a circular solid with varying particle sizes, while TA-Fe@L100 appears as a smooth plate-like solid after freeze-drying (Fig. 4A). The XRD results showed that TA-Fe@L100 existed in an amorphous form (Fig. 4B). To evaluate the enteric properties of TA-Fe@L100, we measured its stability in simulated gastric fluid (SGF) at pH 1.4 and simulated intestinal fluid (SIF) at pH 6.8. The SEM results show that TA-Fe@L100 can still maintain a relatively intact morphology under acidic conditions. After incubation in SIF, obvious erosion phenomena can be observed on the surface of TA-Fe@L100, with partial peeling of surface material, concave holes in the center of the block, and overall unevenness (Fig. 4C). At the same time, the presence of nanoparticles with a particle size of approximately 450 nm can be detected in the solution (Fig. 4D). Therefore, enteric coated materials can enhance the stability of formulations under acidic conditions, and TA-Fe@L100 maintains an insoluble state at pH 1.4. After entering the simulated intestinal fluid environment with pH 6.8, the enteric coated material dissolved and released nanoparticles, which could maintain good colloidal stability within 8 h (Fig. 4E).

We further measured the release behavior of iron ions from TA-Fe NPs and TA-Fe@L100 at different pH values. Consistent with the above results, TA-Fe NPs exhibit structural stability under neutral conditions, but a sudden release of iron ions occurs at pH 1.4 (Fig. 4F), indicating the acid sensitive dissociation properties of the coordination structure. After encapsulating TA-Fe in enteric coated materials, the stability of the formulation was significantly improved, and the release in simulated gastric juice decreased from 81 % to 18 % (Fig. 4G). Therefore, enteric coating can effectively protect nanoparticles from the drastic pH changes in simulated gastrointestinal fluid and release nanoparticles in the small intestine, which is beneficial for the effective absorption of nanoparticles in the small intestine.

### 3.5 Therapeutic effect of TA-Fe@L100 in a mouse IDA model

**Fig. 5:**
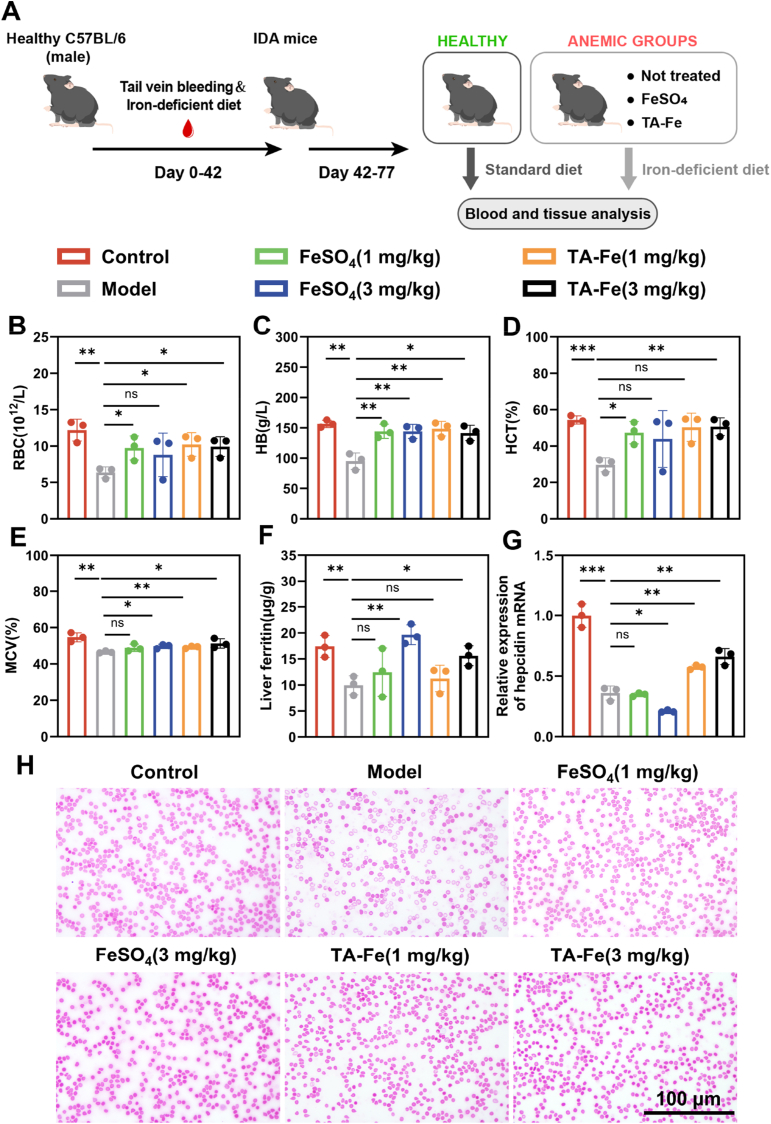
(A) Schematic diagram of IDA induction and treatment regimens with various iron supplements. (B) Red blood cell (RBC) count, (C) Hemoglobin (HB) levels, (D) Hematocrit (HCT) levels, and (E) Mean corpuscular volume (MCV) of red blood cells in mice treated with different iron supplements (n = 3). (F) Liver ferritin content in mice treated with different iron supplements (n = 3). (G) Hepcidin mRNA expression levels in mice treated with different iron supplements (n = 3). (H) Blood smear images of mice from different treatment groups.

Following the formulation and cell biology evaluations, we proceeded to assess the pharmacological efficacy of TA-Fe@L100 in an animal model of iron deficiency anemia (IDA) (Fig. 5A). Four-week-old male C57BL/6 mice were used to establish the IDA model through an iron-deficient diet and tail vein bloodletting. The hallmark of IDA is a decrease in hemoglobin (HB) levels, and after 42 days, the model group mice exhibited HB levels below 125 g/L [41], confirming the successful induction of IDA. The model group was then randomly divided into multiple treatment groups and administered different oral doses for 5 consecutive weeks: FeSO_4_ (1 mg/kg), FeSO_4_ (3 mg/kg), TA-Fe@L100 (1 mg/kg), and TA-Fe@L100 (3 mg/kg) (doses based on iron content, hereafter referred to as TA-Fe).

All treatment groups showed significant therapeutic effects compared to the model group, with notable improvements in hematological indicators, including red blood cell count (RBC), hemoglobin concentration (HB), hematocrit (HCT), and mean corpuscular volume (MCV) (Fig. 5B–E). Dose-dependent increases in these parameters were observed, reflecting the beneficial effects of the treatments. The increase in RBC count and HB concentration demonstrates enhanced erythropoiesis, while the increase in HCT supports an elevation in total blood volume. Additionally, the increase in MCV suggests improved maturation of red blood cells. These hematological improvements highlight that the therapeutic efficacy of TA-Fe@L100 is comparable to that of traditional FeSO_4_.

The liver, as the main storage site for iron, plays a critical role in iron homeostasis. Ferritin is the primary protein responsible for iron storage in the liver. At higher doses (3 mg/kg FeSO_4_ and TA-Fe@L100), ferritin levels in the liver significantly increased (Fig. 5F), indicating effective replenishment of iron reserves in the body.

Hepcidin, a peptide hormone produced by the liver, regulates iron absorption, storage, and release, maintaining systemic iron balance [42]. Under iron deficiency, hepcidin expression is downregulated to increase iron availability. In our study, qPCR analysis of mRNA expression related to iron metabolism revealed a marked decrease in hepcidin expression in the model group, with no significant change in the FeSO_4_-treated groups. However, in the TA-Fe@L100 treatment groups (1 mg/kg and 3 mg/kg), a trend of upregulation in hepcidin mRNA expression was observed, indicating that TA-Fe@L100 treatment effectively restores iron levels and maintains iron homeostasis (Fig. 5G). The upregulated mRNA expression of hepcidin in the TA-Fe@L100 group may indicate enhanced iron uptake efficiency of the nanoparticles, which increases the local iron load in hepatocytes, leading to more active iron metabolism.

Blood smear analysis revealed that red blood cells from the normal group displayed a typical biconcave disk shape and were uniformly distributed (Fig. 5H). In contrast, the model group showed an increased number of hypochromic and microcytic red blood cells, indicating impaired erythropoiesis and iron deficiency. After treatment with TA-Fe@L100, red blood cells in all treatment groups gradually returned to normal morphology, further demonstrating the therapeutic potential of TA-Fe@L100 in correcting iron deficiency anemia.

### 3.6 Regulatory effect of TA-Fe@L100 on gut microbiota in mice

Iron is an essential nutrient for intestinal bacteria, and iron deficiency can significantly impact the composition and diversity of the gut microbiota in iron deficiency anemia (IDA) patients, inhibiting the growth of iron-dependent bacterial species [43,44]. To evaluate the potential regulatory effects of TA-Fe@L100 on the gut microbiota in IDA, we collected fecal samples from each group of mice and analyzed their gut microbiota profiles using 16S rRNA sequencing.

**Fig. 6:**
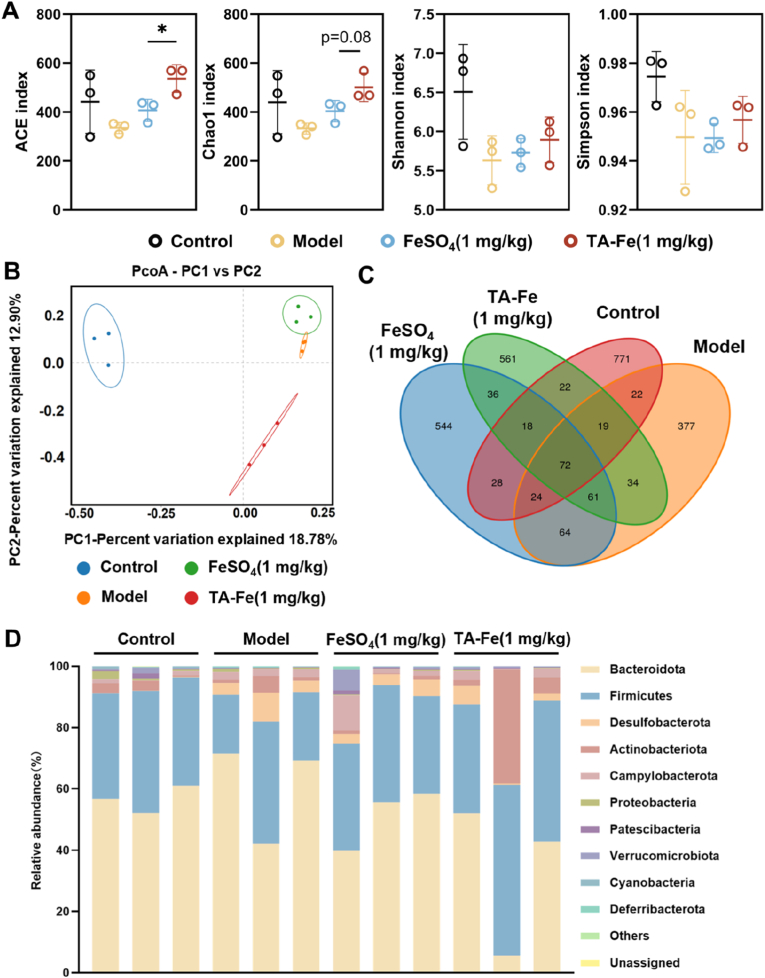
16S rRNA sequencing analysis showing the positive regulatory effects of TA-Fe@L100 (abbreviated as TA-Fe) on the gut microbiota: (A) Alpha diversity indices (ACE and Chao1) showing microbial richness and diversity (n = 3). (B) Principal Coordinate Analysis (PcoA) of β-diversity, reflecting the differences in gut microbiota composition between groups (n = 3). (C) Venn diagram showing the overlap of microbial species between treatment groups. (D) Abundance composition of gut microbiota at the phylum level (n = 3).

The richness and diversity of the gut microbiota were assessed by alpha diversity analysis (Fig. 6A). After TA-Fe@L100 treatment, both ACE and Chao1 indices increased, suggesting that nanoparticle-based therapy has a more favorable effect on the gut microbiota compared to free iron supplementation. However, the Shannon and Simpson indices did not show significant differences. To examine the overall changes in the species composition, we performed β-diversity analysis using Principal Coordinate Analysis (PcoA). The PcoA results showed a clear separation between the healthy and IDA model groups, indicating a distinct difference in gut microbiota composition (Fig. 6B). Although iron supplementation was provided, the microbiota structure in the FeSO_4_-treated group remained similar to that of the model group, suggesting that traditional iron therapy may not effectively restore microbiota balance, potentially due to gastrointestinal side effects (vide infra). In contrast, the microbiota structure in the TA-Fe@L100-treated group showed a significant deviation from the model group, moving closer to that of healthy mice.

A Venn diagram (Fig. 6C) revealed that the number of shared microbial species between the TA-Fe@L100-treated and model groups was lower than that between the FeSO_4_-treated and model groups, implying that TA-Fe@L100 may help restore the gut microbiota to a more normal, healthy state. The abundance of the top 10 bacterial phyla and classes was also analyzed (Fig. 6D and Fig. S5). Firmicutes, a phylum crucial for maintaining intestinal barrier function, slightly increased in both the FeSO_4_ and TA-Fe@L100 treatment groups compared to the model group, suggesting that iron supplementation may help improve intestinal health and alleviate inflammation by promoting beneficial Firmicutes bacteria.

**Fig. 7:**
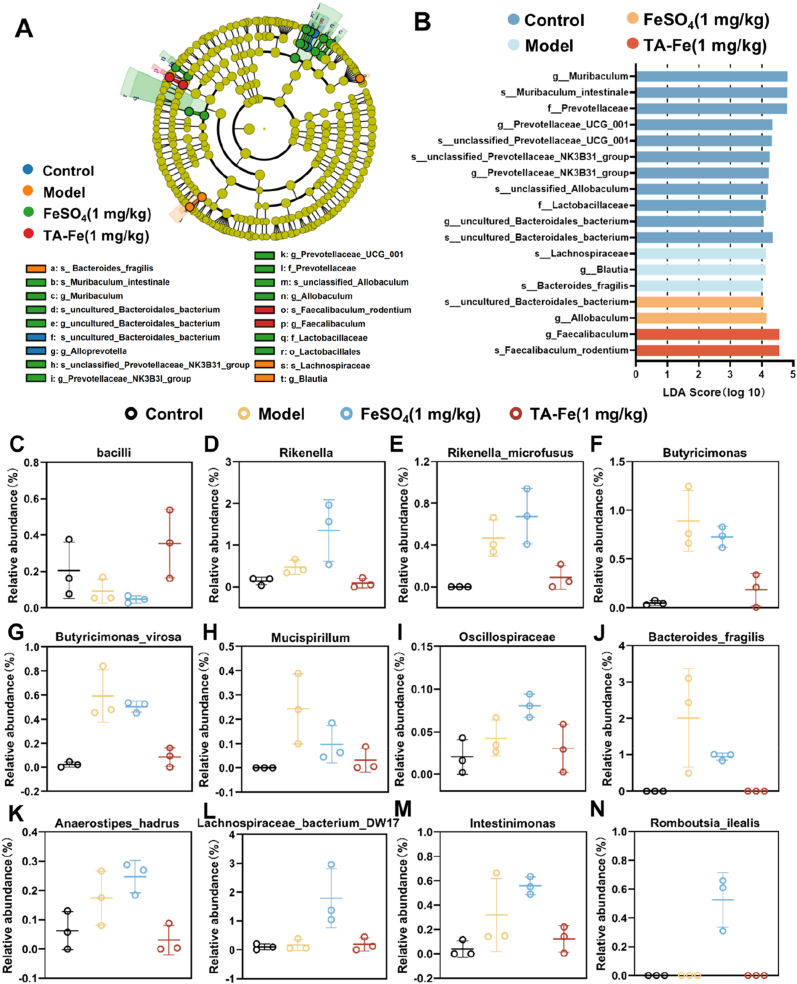
16S rRNA sequencing analysis showing the effects of TA-Fe@L100 (abbreviated as TA-Fe) on gut microbiota: (A) LEfSe taxonomy tree showing phylogenetic differences among the treatment groups. (B) LDA score plot showing the relationship between microbial species and the treatment group. (C–N) Relative abundance changes in specific microbial taxa after treatment with different iron supplements (n = 3).

LEfSe (Linear Discriminant Analysis Effect Size) analysis was performed to identify phylogenetic differences between microbial communities in the different treatment groups (Fig. 7A). The LDA score plot (Fig. 7B) was used to quantify and visualize the association between each microorganism with significant differences and the experimental treatment. Higher LDA scores correspond to stronger associations between specific microbial species and the treatment group. The results indicated that microorganisms such as *g_Muribaculum* and *f_Prevotellaceae* were enriched in the intestines of healthy mice, while *s_Lachnospiraceae* and *g_Blautia* were enriched in the intestines of mice with IDA. *S_uncultured_Bacteroidales_macterium* and *d_Allobaculum* were enriched in the intestines of FeSO_4_-treated mice, while *d_Faecalibaculum* and *s_Faecalibaculum_rodentium* were enriched in the intestines of TA-Fe@L100-treated mice.

Further analysis of the differences in gut microbiota among the treatment groups revealed significant changes in the abundance of several key bacterial taxa. The Bacilli class, a taxonomic group within the Firmicutes phylum, increased in abundance in the TA-Fe@L100-treated group, indicating potential improvements in intestinal health (Fig. 7C). Additionally, microbial communities associated with oxidative stress regulation, such as *Rikenella* [45,46] and *Butyricimonas* [47,48], which are known to have pro-inflammatory or pro-oxidative effects, showed significant increases in the model group. These communities were restored to normal levels following TA-Fe@L100 treatment (Fig. 7D–H).

We also found that some microbial species associated with short-chain fatty acid (SCFA) production, such as *Oscillospiraceae*, *Bacteroides_fragilis*, *Anaerostipes_cadrus*, and *Lachnospiraceae_bacterium-DW17*, showed dysbiosis in the model and FeSO_4_ groups. These SCFA-producing microbes are linked to inflammatory bowel disease and chronic inflammation [[49], [50], [51], [52], [53]]. However, after treatment with TA-Fe@L100, the abundance of these microbiota returned to normal levels (Fig. 7I–L). Furthermore, *Intestinimonas* and *Romboutsia_ilealis* [54,55], which are associated with obesity-related diseases, showed changes in abundance in both the model and FeSO_4_ groups. These changes may indicate an increased risk of metabolic diseases associated with iron deficiency or intestinal oxidative stress. Treatment with TA-Fe@L100 restored their abundance to normal levels (Fig. 7M and N).

In summary, the gut microbiota of mice with iron deficiency anemia exhibits significant imbalances. Traditional iron supplementation (FeSO_4_) did not effectively improve the gut microbiota composition, and oxidative stress caused by free iron may exacerbate these imbalances. In contrast, TA-Fe@L100 treatment effectively restored the microbial communities to normal levels, likely due to its antioxidant properties and reduced intestinal free iron concentration. Overall, TA-Fe@L100 demonstrates a significant regulatory effect on gut microbiota at multiple taxonomic levels, including class, family, genus, and species.

### 3.7 Alleviating gastrointestinal side effects of iron supplements by TA-Fe@L100

**Fig. 8:**
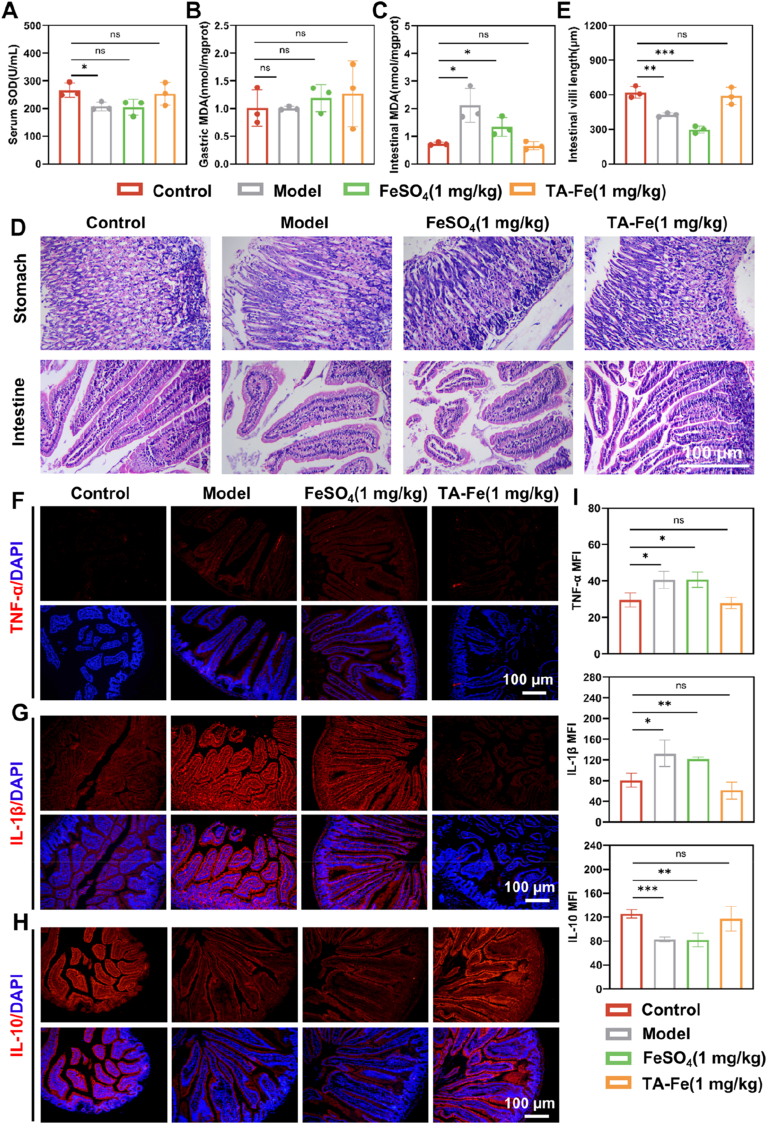
The gastrointestinal side effect alleviation of TA-Fe@L100 (abbreviated as TA-Fe) treatment: (A) Plasma SOD content in mice treated with different iron supplements (n = 3). (B) Gastric MDA content in mice after treatment with different iron supplements (n = 3). (C) MDA content in the small intestine after treatment with different iron supplements (n = 3). (D) Length of small intestinal villi in mice after treatment with different iron supplements (n = 3). (E) H&E staining of stomach and small intestine sections showing tissue damage and repair. (F–H) Immunofluorescence staining of TNF-α, IL-1β, and IL-10 in the small intestine of mice. (I) Semi-quantitative analysis of TNF-α, IL-1β, and IL-10 expression levels (n = 3).

After confirming the therapeutic effects of TA-Fe@L100 in IDA, we further investigated its potential to alleviate gastrointestinal oxidative stress and inflammation compared to traditional iron supplements under long-term administration. Superoxide dismutase (SOD) activity is widely used as an indicator of cellular antioxidant capacity. In the model group, serum SOD levels were significantly decreased compared to the normal group, indicating a weakened antioxidant defense in the body due to anemia. Treatment with FeSO_4_ did not alleviate this reduction, while treatment with TA-Fe@L100 restored SOD levels to near normal levels, suggesting its antioxidant potential (Fig. 8A).

Malondialdehyde (MDA), a byproduct of lipid peroxidation, reflects the extent of oxidative stress and free radical production. In the stomach, MDA levels did not show significant changes after treatment (Fig. 8B). However, in the small intestine, the model group exhibited pronounced oxidative stress damage, as indicated by elevated MDA levels in the mucosa. While FeSO_4_ treatment only slightly reduced MDA levels without statistical significance, TA-Fe@L100 treatment significantly lowered MDA levels to the same level as the normal group, highlighting its capacity to mitigate oxidative stress in the small intestine (Fig. 8C). This effect is likely due to the antioxidant properties of TA in the nanoparticle formulation, as well as the protection provided by the nanoparticle's controlled iron release, which prevents oxidative damage from free iron exposure.

Histological analysis of gastric and intestinal tissues further corroborated these findings. Normal gastric tissue showed glandular ducts and a single layer of columnar epithelium with uniform cell size and no atypia (Fig. 8D). In contrast, the model group displayed epithelial shedding, abnormal cell size, congestion, and mild inflammatory cell infiltration. In the FeSO_4_ treatment group, epithelial cells showed atypia, darker chromatin, and spindle-shaped morphologies, with some inflammatory infiltration and significant submucosal edema. After treatment with TA-Fe@L100, gastric tissue exhibited uniform cell size and a restoration of normal epithelial morphology. Similarly, the intestinal epithelium in the model group showed mild proliferation, while the FeSO_4_ group exhibited significant epithelial shedding and disordered tissue structure. TA-Fe@L100 treatment resulted in a consistent cell size and normal tissue phenotype. Additionally, the small intestinal villi length, which was significantly shortened in the model and FeSO_4_ treatment groups, was restored to normal after TA-Fe@L100 treatment (Fig. 8E).

To assess local inflammation in the small intestine, immunofluorescence staining was used to measure the expression of pro-inflammatory cytokines (TNF-α, IL-1β) and the anti-inflammatory cytokine IL-10 (Fig. 8F–I). In the model group, TNF-α and IL-1β levels were significantly elevated, while IL-10 levels were notably decreased, indicating an inflammatory response due to iron deficiency. Although FeSO_4_ treatment alleviated iron deficiency symptoms, the accumulation of metal ions in the intestine led to ROS production and exacerbated local inflammation. This resulted in limited anti-inflammatory effects. In contrast, TA-Fe@L100 treatment effectively suppressed the production of pro-inflammatory cytokines and promoted the release of IL-10, resulting in a significant reduction in intestinal inflammation.

These findings suggest that TA-Fe@L100 not only provides effective iron supplementation but also reduces oxidative stress and inflammation in the gastrointestinal tract, promoting tissue repair and alleviating the side effects typically associated with oral iron supplements. Compared to traditional iron supplements, TA-Fe@L100 shows superior efficacy in mitigating gastrointestinal side effects while enhancing iron therapy.

### 3.8 In vivo safety evaluation of TA-Fe@L100

**Fig. 9:**
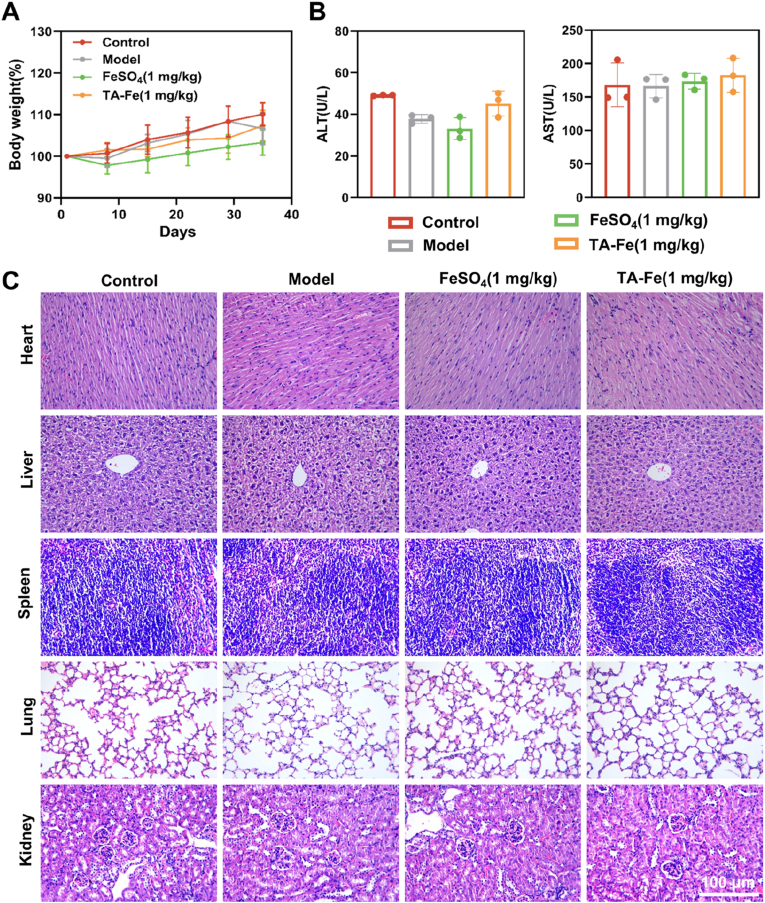
(A) Weight change of mice treated with different iron supplements during the administration period (n = 12). (B) Plasma ALT and AST levels in mice treated with different iron supplements (n = 3). (C) H&E staining of major organ tissues (heart, liver, spleen, lungs, kidneys) in mice after treatment with different iron supplements.

To assess the safety of TA-Fe@L100, we conducted an in vivo evaluation focusing on weight monitoring, biochemical index detection, and histopathological analysis. Throughout the administration period, no significant differences in weight gain were observed among the treatment groups (Fig. 9A). However, the FeSO_4_ group exhibited slower weight gain compared to the other groups, likely due to gastrointestinal side effects associated with free iron supplementation. Biochemical analysis of liver function, including measurements of alanine aminotransferase (ALT) and aspartate aminotransferase (AST), revealed no significant differences between the treatment groups (Fig. 9B). This indicates that long-term oral administration of TA-Fe@L100 does not adversely affect liver function. Histopathological examination of major organs, including the heart, liver, spleen, lungs, and kidneys, showed no significant changes or signs of toxicity following treatment (Fig. 9C). The tissues exhibited normal architecture with no evidence of damage or inflammation. Collectively, these results demonstrate the safety of long-term oral administration of TA-Fe@L100, with no significant adverse effects on body weight, liver function, or major organ histology.

## 4 Conclusion

In conclusion, TA-Fe@L100, a novel nanoparticle-based iron supplement, offers significant advantages over traditional iron supplements in the treatment of IDA. By leveraging the properties of metal-polyphenol networks (MPNs), TA-Fe NPs not only enhance the chemical stability of iron, avoid the negative of effect of food, but also provide antioxidant and anti-inflammatory benefits, addressing the key limitations of conventional oral iron therapies. The enteric coating (TA-Fe@L100) ensures stability during gastrointestinal transit, while the polyphenolic TA component mitigates oxidative stress and local inflammation, thereby alleviating common side effects such as gastrointestinal irritation. Our in vivo studies demonstrate that TA-Fe@L100 effectively improves hematological parameters, organ iron storage, and gut microbiota balance, without the adverse effects associated with traditional iron salts. Given its simplicity in preparation, low cost, and superior efficacy, TA-Fe@L100 holds great promise as a new oral iron supplement for the treatment of IDA. This approach could provide a more effective, patient-friendly alternative to current iron therapies, contributing to improved treatment outcomes and patient compliance.

## CRediT authorship contribution statement

**Ying Yao:** Writing – review & editing, Writing – original draft, Formal analysis, Data curation. **Yuanzheng Chen:** Formal analysis, Data curation. **Jie Fu:** Formal analysis, Data curation. **Jinsong Ding:** Formal analysis, Data curation. **Wenhu Zhou:** Writing – review & editing, Writing – original draft. **Xinyi Chen:** Writing – review & editing, Data curation. **Xiuping Wan:** Writing – review & editing, Data curation.

## Declaration of competing interest

The authors declare that they have no known competing financial interests or personal relationships that could have appeared to influence the work reported in this paper.

## Data Availability

Data will be made available on request.
