# Supplementary material for: A metal-polyphenol network-based iron supplement with improved stability and reduced gastrointestinal toxicity for iron deficiency anemia therapy
Source: Mater Today Bio. 2025 Feb 20;31:101598. doi: 10.1016/j.mtbio.2025.101598 (PMC11894331; doi:10.1016/j.mtbio.2025.101598)
Supplement: Multimedia component 1 [file mmc1.docx]

**Supporting Information**

**A Metal-Polyphenol Network-Based Iron Supplement with Improved Stability and Reduced Gastrointestinal Toxicity for Iron Deficiency Anemia Therapy**

Ying Yao^1+^, Yuanzheng Chen^1+^, Jie Fu^1^, Jinsong Ding^1^, Wenhu Zhou*^1^, Xinyi Chen*^2^, Xiuping Wan*^3^

1. Xiangya School of Pharmaceutical Sciences, Central South University, Changsha, Hunan, 410013, China

2. Yongkang First People's Hospital of Wenzhou Medical University, Jinhua, 321300, China

3. Department of Gastroenterology, The Quzhou Affiliated Hospital of Wenzhou Medical University, Quzhou People’s Hospital, Quzhou, 324000, China

[^+^] These authors contributed equally to this work.

E-mail: [wanxiuping@wmu.edu.cn](mailto:wanxiuping@wmu.edu.cn);

[chenxinyi0428@163.com](mailto:chenxinyi0428@163.com);

[zhouwenhuyaoji@163.com](mailto:zhouwenhuyaoji@163.com)


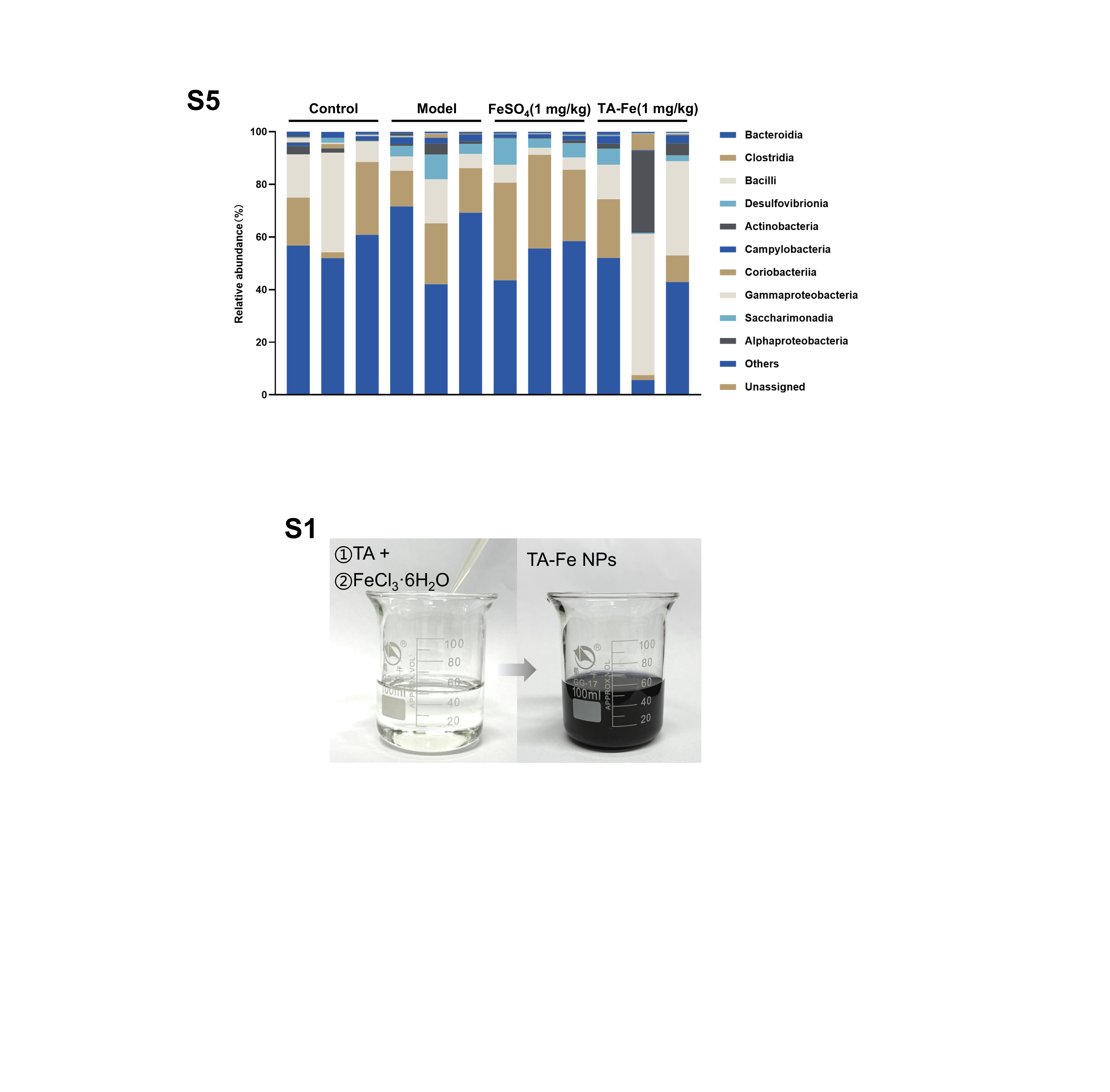


**Fig S1.** Preparation of nanoparticles. Add light yellow TA and FeCl_3_·6H_2_O solution to ultrapure water, then the solution turns blue black.

**Fig S2.** Molecular dynamics simulation of the interaction between TA and Fe^3+^. (A) Schematic diagram of REST and cluster analysis of TA molecules. (B) Interaction of TA complex with Fe^3+^. (C) Binding energy and binding mode of TA complex and TA-Fe^3+^ complex. (D) LUMO and HOMO molecular orbital analysis of the interaction between TA and Fe^3+^.

**Fig S3.** (A) UV spectra, (B) kinetic curves, and (C) scavenging rates (n=3) of DPPH radicals scavenged by TA-Fe NPs at different concentrations. (D) UV spectra, (E) kinetic curves, and (F) clearance (n=3) of ABTS radical scavenging by TA-Fe NPs at different concentrations. (G) UV spectra and (H) clearance of •OH by TA-Fe NPs at different concentrations (n=3). (I) UV spectra and (J) clearance of O_2_^-^• for TA-Fe NPs at different concentrations (n=3).

**Fig S4.** (A) Sample appearance and (B) particle size plot (n=3) of TA-Fe NPs under different pH conditions as a function of time.


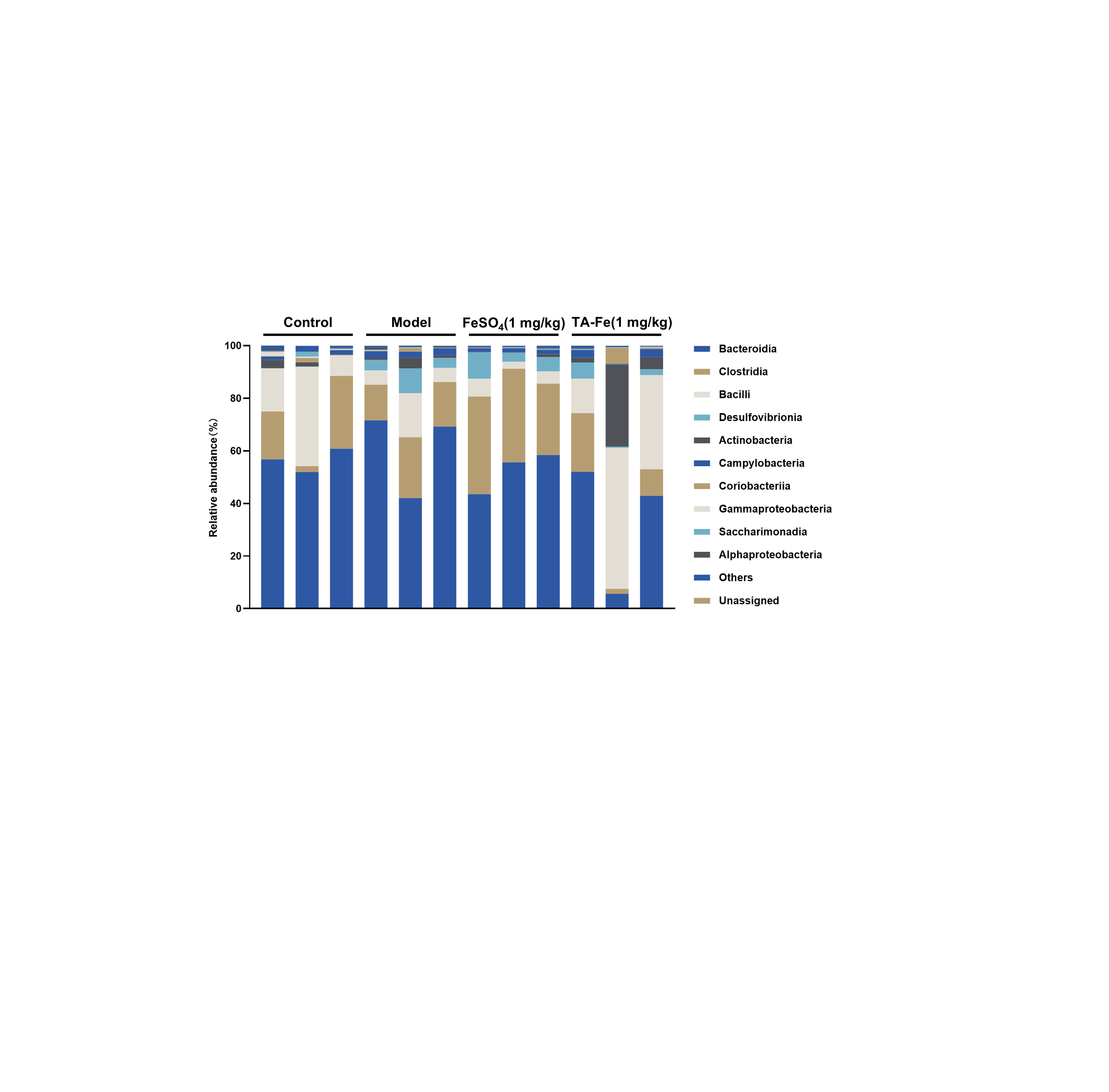


**Fig S5.** Species richness of gut microbiota at the class level.
